# Reply to: Higher PEEP in intubated COVID-19-associated ARDS patients? We are not sure

**DOI:** 10.1186/s13054-022-04262-z

**Published:** 2022-12-14

**Authors:** Peter Somhorst, Philip van der Zee, Henrik Endeman, Diederik Gommers

**Affiliations:** Department of Adult Intensive Care, Erasmus Medical Center, Doctor Molewaterplein 40, 3015 GD Rotterdam, The Netherlands

Dear editor,

We would like to thank Dr. Yaroshetskiy and colleagues for their interesting comments on our manuscript. The commenters underline the importance of individualizing PEEP based on the individual physiology and the importance of BMI in choosing the PEEP level.

We would like to point out that the reported PaO_2_/FiO_2_ ratio was achieved with invasive mechanical ventilation with significant PEEP (17.0 [16.0–19.0] cmH_2_O). The lowest recorded PaO_2_/FiO_2_ ratio (immediately after intubation) was 121 [84–180] mmHg. The commenters refer to Perkins et al. [1], who showed only a significant but small decrease in intubation rate when using CPAP and no difference when using HFNO compared to conventional oxygen therapy. We therefore do not agree with the commenters that a significant portion of this cohort could have been ventilated with CPAP or HFOT alone.

Electrical Impedance Tomography showed significant recruitability, especially in the PEEP_higher_ group. This recruitment is not reflected in an increase in compliance. As we previously described we accepted slightly more overdistention than alveolar collapse, which counteracts the increase in compliance due to recruitment. Although optimal compliance is often used as a measure of recruitment, we have no indication the optimal compliance coincides with the best trade-off between recruitment and overdistention. We also point out driving pressure was unchanged due to the pressure control mechanical ventilation. However, tidal volume increased from 6.1 (1.2) to 6.6 (1.3) mL/kg. The commenters suggest recruitment must be accompanied by an increase in PaO_2_/FiO_2_ ratio, overlooking the precarious balance between recruitment and overdistention and the potential of perfusion impairment. We do not agree with the authors that PaO_2_/FiO_2_ ratio is a proper measure for recruitment.

We agree that the method of estimating local compliance using EIT is not without limitations. We use changes in local impedance variation to estimate collapse and overdistention, making the patient their own control. We are not sure what the commenters refer to when they suggest an increase in strain can occur without change in volume. Strain is defined as a the ratio between a change in lung volume—due to either PEEP or tidal ventilation—and the functional residual capacity [2]. Stress can be increased during spontaneous efforts [3], but in our study spontaneous efforts were prevented with high levels of sedation or neuromuscular blockage if necessary.

We have not provided any guidance or recommendations on how to set PEEP. The PEEP/FiO_2_ tables from the ARDSNet trials are most widely used, but no optimal strategy has been identified [4]. Larger outcome studies are required, which not only include EIT measurements, but also esophageal manometry [5] and other modalities. We would like to thank the commenters for the chance to reiterate our: personalization of mechanical ventilation based on the individual physiology is crucial for patients with (COVID-19-related) ARDS.

## Data Availability

Not applicable.
